# Supplementary material for: Hyaluronidase-Responsive Mesoporous Silica Nanoparticles with Dual-Imaging and Dual-Target Function
Source: Cancers (Basel). 2019 May 20;11(5):697. doi: 10.3390/cancers11050697 (PMC6562767; doi:10.3390/cancers11050697)
Supplement: Supplementary file 1 [file cancers-11-00697-s001.pdf]

Supplementary Materials

Hyaluronidase-Responsive Mesoporous Silica Nanoparticles with Dual-Imaging and Dual-Target Function

Table S1. Hydrodynamic size and zeta potential for MSN-EuGd and functionalized MSN-EuGd.

	MSN-EuGd	MSN-EuGd-NH	MSN-EuGd-TAT	MSN-EuGd-TAT-HA
Zetapotential (mV)	−14.5	−10.5	4.08	−17.3
Particle size (nm)	271.2	279.9	381.1	457.5

Table S2. ICP-MS analyze of MSN and MSN-EuGd.

Sample	Eu	Gd
MSN	0%	0%
MSN-EuGd	1.06%	1.02%

Table S3. TGA for the MSN-EuGd@CPT-TAT-PEG-FA.

MSN-EuGd	-NH_2	-TAT	@CPT	-HA
Weight (%)	11.44	2.56	1.5	6.15
mg/g	129.17	26.27	15.22	65.53

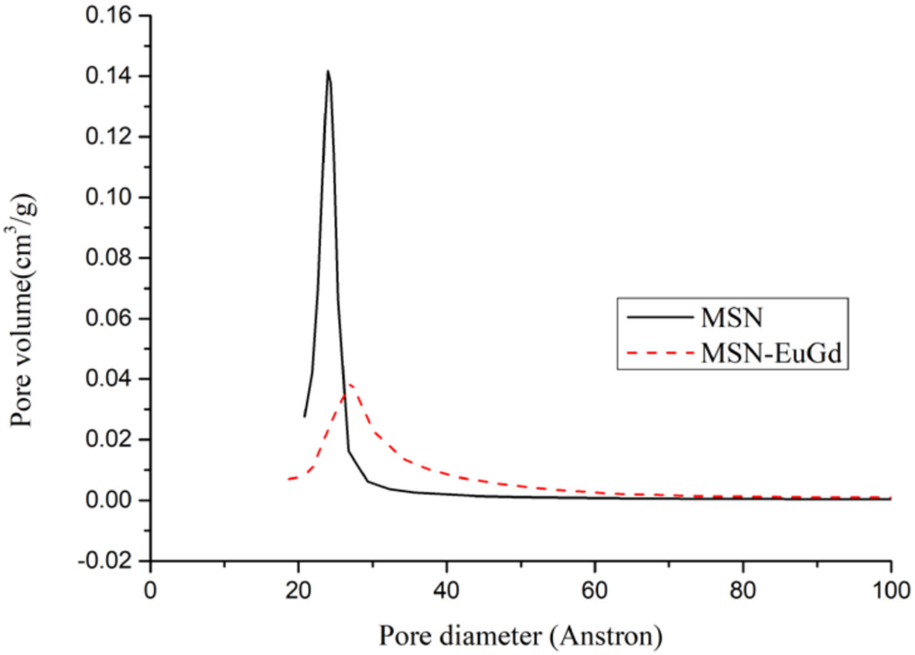

Figure S1. BJH pore size distribution of MSN and MSN-EuGd.

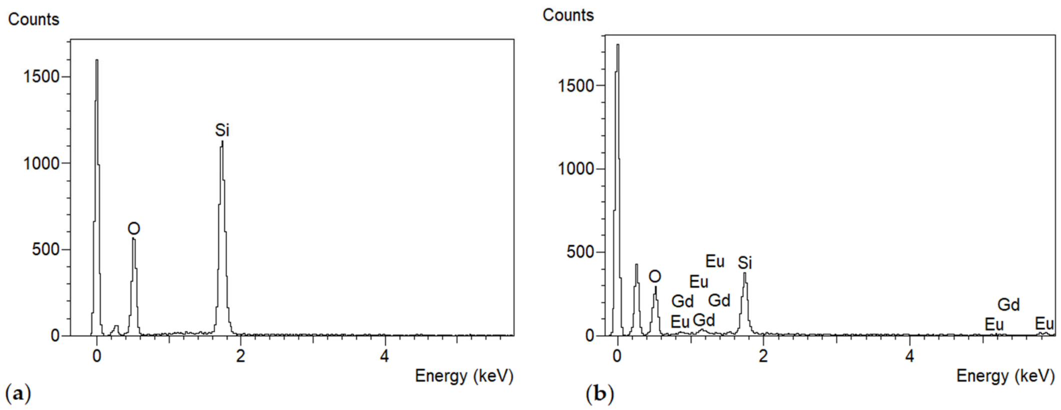

Figure S2. EDX spectral analyses of the (**a**) MSN and (**b**) MSN-EuGd.

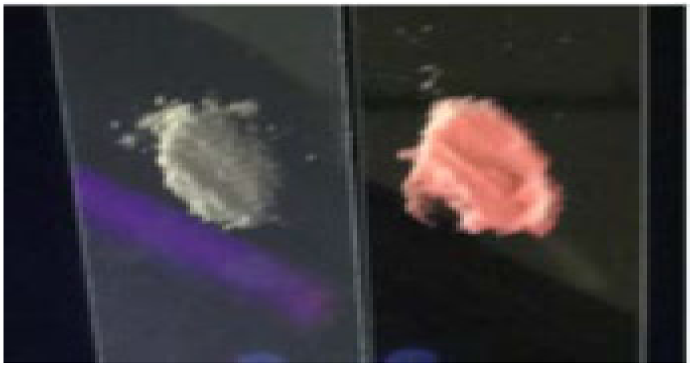

Figure S3. MSN and MSN-EuGd powder taken under illumination by a 254 nm UV lamp.

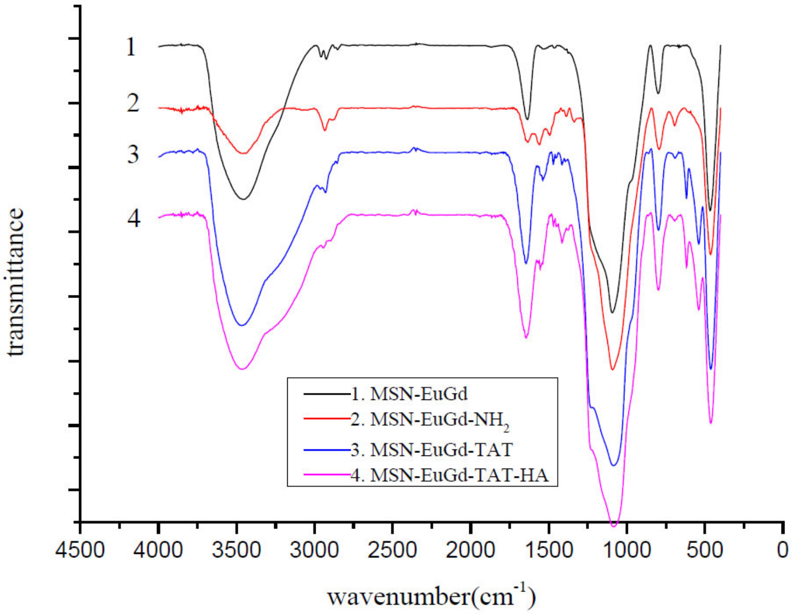

Figure S4. FTIR spectrum of MSN-EuGd and functionized MSN-EuGd.

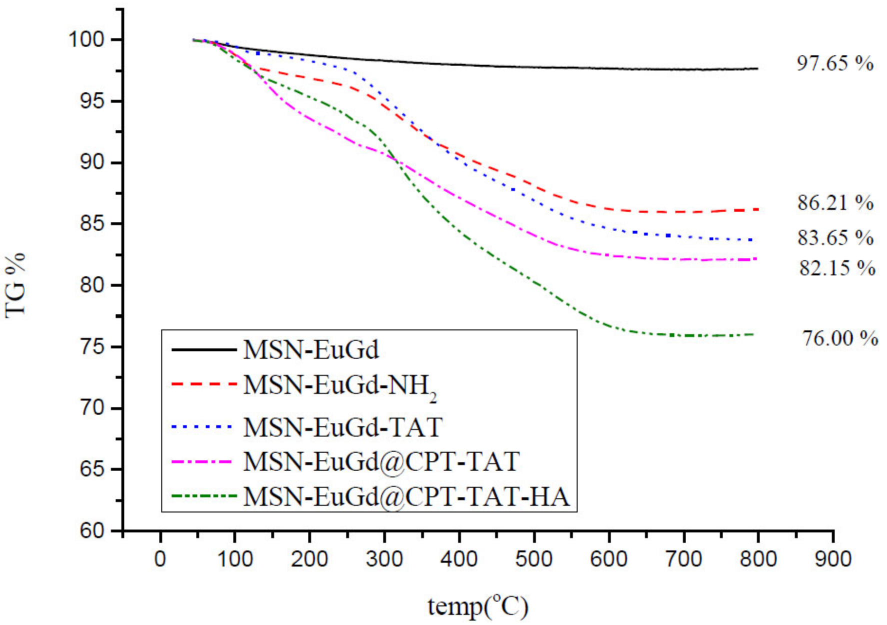

Figure S5. TGA patterns for the MSN-EuGd and functionized MSN-EuGd.
